# Risk Factors for Brain Metastases in Patients with Renal Cell Carcinoma

**DOI:** 10.1155/2020/6836234

**Published:** 2020-03-09

**Authors:** Zhi-Bin Ke, Shao-Hao Chen, Ye-Hui Chen, Yu-Peng Wu, Fei Lin, Xue-Yi Xue, Qing-Shui Zheng, Ning Xu, Yong Wei

**Affiliations:** Department of Urology, The First Affiliated Hospital of Fujian Medical University, Fuzhou 350005, China

## Abstract

**Background:**

Patients with brain metastases (BM) from renal cell carcinoma (RCC) were considered to experience a poor prognosis. However, there is little knowledge on the risk factors for BM from RCC at diagnosis. This study was aimed at exploring the risk factors for patients with BM from RCC and the interaction among these risk factors.

**Methods:**

A total of 38759 cases of RCC were identified from the Surveillance, Epidemiology, and End Results (SEER) database. Risk factors for BM from RCC were evaluated by univariate and multivariate logistic regression analyses. Interaction effect between age and tumor size was tested.

**Results:**

There was a significant difference in univariate analysis, including T stage, tumor size, grades III and IV, lymph node metastasis, bone metastasis, liver metastasis, lung metastasis, and surgery. There was a significant difference in multivariate analysis, including age, T stage, tumor size < 10 cm, grade IV, lymph node metastasis, bone metastasis, lung metastasis, and surgery. Patients older than 70 had 0.653-fold lower risk of developing BM compared with those younger than 70. Patients with tumor size ≥ 4 cm and <10 cm had higher risk of developing BM compared with those < 4 cm. The larger the tumor size, the higher the incidence of BM from RCC in those whose tumor size was less than 10 cm. An interaction test between the tumor size and age on brain metastasis was statistically significant in the crude analysis (*P* = 0.0114) and model II analysis (*P* = 0.0114) and model II analysis (*P* = 0.0114) and model II analysis (

**Conclusion:**

Both tumor size and age were independent risk factors for brain metastases in patients with RCC. The impact of age on the risk of developing BM from RCC was limited to patients with tumor size ≥ 7 cm. Patients with a larger tumor size and younger age might have the higher risk of developing BM at diagnosis of RCC.

## 1. Introduction

The incidence of renal cell carcinoma (RCC) has continued to increase recently, most of whom were localized [1, 2]. However, approximately 30% of nonmetastatic RCC would progress to metastatic disease after definitive treatment [3]. Nonmetastatic RCC patients developed brain metastases (BM) in 2.4% of cases [1]. It is estimated that incidence proportions of BM from RCC at diagnosis was 6.5% [1, 4]. Patients with BM from RCC were considered to experience a poor prognosis [5]. In the past 20 years, the incidence of BM from RCC was reported to increase significantly [6, 7]. Understanding the risk factors of developing BM is important to diagnosis, treatment, prevention, and counseling in patients with BM from RCC. However, there was little study investigating the risk factors of patients with BM from RCC. This study is aimed at determining the risk factors for patients with BM from RCC and testing the interaction among these risk factors.

## 2. Methods

### 2.1. Data Source

The Surveillance, Epidemiology, and End Results (SEER) database includes information on demographics, cancer incidence, and survival outcomes from population-based registries for approximately 30% of the US population. Data of this study were obtained from the SEER program of the National Cancer Institute using the SEER∗Stat software (version 8.3.5). Since all information from the SEER database has been deidentified and no personal identifying information was used in this analysis, informed consent is not required for use of the SEER data.

### 2.2. Statistical Analysis

Statistical analysis was performed using SPSS 21 software. Categorical data were presented as frequency (%) and analyzed by the chi-squared test or Fisher's test. Continuous data that were normally distributed were represented as mean ± standard deviation. Univariate and multivariate logistic regression analyses were used to determine risk factors of BM from RCC. *P* < 0.05 was considered to be statistically significant. Besides, interactions between age and tumor size were tested.

## 3. Results

**Figure 1:**
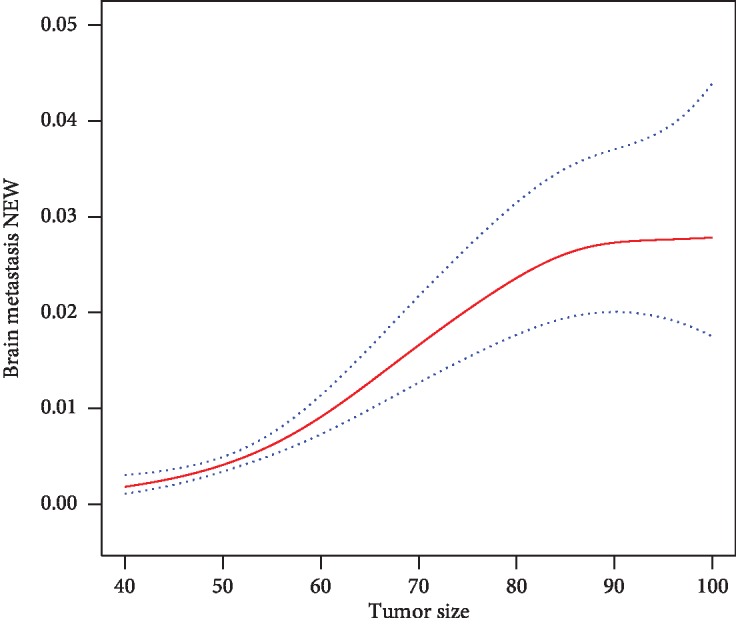
Correlation between tumor volume and incidence of brain metastasis in RCC patients with tumor size < 10 cm.

We finally included 38759 cases of RCC.  Figure 1 reveals that the larger the tumor size, the higher the incidence of brain metastases from RCC in patients whose tumor size was less than 10 cm.

Baseline characteristics of participants are showed in Table 1. The participants were classified into 3 groups by tumor size: tumor size < 7 cm (*n* = 28793); 7 cm ≤ tumor size < 10 cm (*n* = 5804); and tumor size ≥ 10 cm (*n* = 4162). There was a significant difference between these three groups, including tumor size, age, grade, T stage, N stage, M stage, bone metastasis, brain metastasis, liver metastasis, lung metastasis, insurance, Fuhrman grade, surgery, and race, with the exception of marital status. There were significant differences in the incidence of brain metastases among the three groups (0.205% vs. 1.568% vs. 2.427%, *P* < 0.001).

As showed in Table 2, there was a significant difference in univariate analysis, including T stage, tumor size, grades III and IV, lymph node metastasis, bone metastasis, liver metastasis, lung metastasis, and surgery. There was a significant difference in multivariate analysis, including age, T stage, tumor size < 10 cm, grade IV, lymph node metastasis, bone metastasis, lung metastasis, and surgery. Patients older than 70 had 0.653-fold lower risk of developing BM compared with those younger than 70. Patients with tumor size ≥ 4 cm and <7 cm had 2.270-fold higher risk of developing BM compared with those < 4 cm. Patients with tumor size ≥ 7 cm and <10 cm had 2.360-fold higher risk of developing BM compared with those < 4 cm. It was significantly increased in the risk of BM for patients whose tumor size was ≥4 cm and <10 cm compared with patients with tumors < 4 cm in diameter. However, while tumor size was 10 cm or larger, the risk of brain metastasis did not increase significantly compared with patients with tumors < 4 cm in diameter.

Table 3 further quantifies the effect of tumor size and age on the risk of developing BM at diagnosis of RCC. Model I was adjusted for bone metastasis, liver metastasis, and lung metastasis. Model II was adjusted for grade, N stage, marital status, race NEW, insurance, and Fuhrman grade. The patients were classified into 6 groups by tumor size and age: group 1 (tumor size < 7 cm and age < 70 years), group 2 (tumor size < 7 cm and age ≥ 70 years), group 3 (7 cm ≤ tumor size < 10 cm and age < 70 years), group 4 (7 cm ≤ tumor size < 10 cm and age ≥ 70 years), group 5 (tumor size ≥ 10 cm and age < 70 years), and group 6 (tumor size ≥ 10 cm and age ≥ 70 years). There was no significant difference between group 1 and group 2 (*P* > 0.05); however, the difference between group 1 and other groups was statistically significant (*P* < 0.05). Interaction effect between tumor size and age on the risk of brain metastasis was statistically significant in the crude analysis (*P* = 0.0114) and model II analysis (*P* = 0.0248) but not in model I analysis (*P* = 0.1136).

## 4. Discussion

Patients with RCC were in connection with a significant higher mortality rate once brain metastases occurred [8]. Median survival of these patients varied merely from 4.1 months to 13.4 months [9]. But the risk factors for BM due to RCC were unelucidated fully. This study explored the risk factors for BM from RCC and investigated the interaction effect between age and tumor size on the risk of BM from RCC for the first time.

In this study, there was a significant difference in univariate analysis, including T stage, tumor size, grades III and IV, lymph node metastasis, bone metastasis, liver metastasis, lung metastasis, and surgery. There was a significant difference in multivariate analysis, including age, T stage, tumor size < 10 cm, grade IV, lymph node metastasis, bone metastasis, lung metastasis, and surgery. These results demonstrated that younger age, higher T stage, tumor size, higher grade, lymph node or bone or lung metastasis, and nonsurgical treatment were risk factors for BM from RCC. Patients with higher T stage, higher grade, and lymph node or bone or lung metastasis had a higher risk of developing BM from RCC. Besides, the risk of BM was significantly increased in RCC patients without partial/radical nephrectomy, indicating that it is of vital importance for RCC patients to receive surgical treatment of the primary lesion.

This study identified younger age as a risk factor for developing brain metastases, which was also verified in different types of cancer but not in RCC. Ji et al. [10] suggested that age ≤ 60 years was an independent risk factor for BM of patients with stage III locally advanced non-small-cell lung cancer. Korkmaz et al. [11] found that it was easier for patients younger than 65 years to develop hippocampal metastasis. Ma et al. [12] identified age ≤ 53 years as a high-risk factor for developing BM in patients with EGFR-mutated advanced lung adenocarcinoma. Warren et al. [13] found that there was a lower probability of BM in inflammatory breast cancer patients with older age at diagnosis. The study conducted by Hung et al. [14] revealed that patients ≤ 35 years tended to develop cerebral lesions in patients with breast cancer. Maurer et al. [15] showed that age ≤ 40 years was a risk factor for BM in patients with HER2-positive breast cancer. Our results demonstrated that younger patients were prone to developing brain metastases, which was similar to previous speculations. Besides, we identified 70 years old as the threshold value. Patients younger than 70 are more likely to develop BM from RCC compared with those older than 70.

Tumor size influences the risk of developing BM from RCC and other types of tumor, which was demonstrated by previous studies. Schovanek et al. [16] suggested that 4.5 cm was the optimal cut-off primary tumor size which can predict development of any metastases from pheochromocytoma/paraganglioma. Maurer et al. [15] identified tumor size > 2 cm as a risk factor for the development of BM in patients with HER2-positive breast cancer. Sun et al. [7] revealed that white/other race, clear cell histology, sarcomatoid differentiation, T2–4 disease, tumor dimension > 10 cm, and N+ disease were risk factors of BM development at RCC diagnosis. Besides, they constructed a risk model, which included tumor size. In their opinion, patients with tumor size > 10 cm were more likely to develop BM from RCC, which is inconsistent with our research. This study showed that patients with tumor size ≥ 4 cm had the higher risk of BM from RCC. There were significant differences in the incidence of brain metastases among the three groups: tumor size < 7 cm; 7 cm ≤ tumor size < 10 cm; and tumor size ≥ 10 cm (0.205% vs. 1.568% vs. 2.427%, *P* < 0.001). Moreover, in those whose tumor size was less than 10 cm, there appeared to be continuously increased risk of BM from RCC with the increase of primary tumor size. However, although the risk of developing BM in patients with tumors size ≥ 10 cm was higher compared with those < 4 cm, it was not statistically significant.

There was a statistically significant interaction between age and tumor size in the crude analysis (*P* = 0.0114) and model II analysis (*P* = 0.0248), demonstrating that the effect of age on the risk of BM from RCC was affected significantly by tumor size. Although previous studies have identified the effect of tumor size on the risk of BM from RCC, our analysis revealed for the first time that age significantly affected the risk of developing BM from RCC and the impact of age was limited to patients with primary tumor size ≥ 7 cm. Tran et al. [17] have also explored the interaction effect between tumor size and age on the prognosis of patients with papillary thyroid carcinoma and found that the impact of tumor size on RFS was limited to patients aged ≥55 years. However, to our knowledge, there are no previous studies analyzing the interaction effect between tumor size and age on the risk of BM from RCC patients. This study suggested that the higher risk of BM from RCC was observed in patients younger than 70 years old and the risk was even higher if his/her tumor size was 7 cm or larger. Besides, the impact of age was limited to patients with primary tumor size ≥ 7 cm.

Our findings, if confirmed, have certain clinical implications. Those patients with a combination of age younger than 70 years old and tumors size ≥ 7 cm experience higher risk of developing brain metastasis. Clinicians should perform brain computed tomography (CT) scans or magnetic resonance imaging (MRI) in patients younger than 70 years old for brain metastasis screening. If his/her tumor size was larger than 7 cm, the need for brain CT or MRI screening was greatly increased.

Given that our study is large population-based, there were several unavoidable limitations that warrant consideration. Firstly, as a result of nonrandomized patient population, selection bias may occur in this study. Secondly, the comorbidities and performance status were not available in the SEER registry. Thirdly, the population of the SEER database sampled only in America may affect the generalizability of this study. Fourthly, theoretically, only if all patients underwent brain imaging, the true incidence of brain metastases could be calculated. However, there was no information on how BM were diagnosed in the SEER database, and we could not exclude those BM patients who were diagnosed by symptoms. Fifthly, the SEER database did not record detailed follow-up data on BM during the course of the disease. Hence, it is unable for us to evaluate how many patients developed BM during the course of the disease.

## 5. Conclusion

Both tumor size and age were independent risk factors for BM in patients with RCC. Patients with a larger tumor size and younger age might have the higher risk of developing BM at diagnosis of RCC. The impact of age on the risk of developing BM from RCC was limited to patients with tumor size ≥ 7 cm. Age appeared not to impact the risk of BM development in patients with a smaller tumor size. Our findings need further investigation.

## Figures and Tables

**Table 1:** Baseline characteristics classified by primary tumor size of renal cell carcinoma.

Tumor size	<7 cm	≥7 cm and <10 cm	≥10 cm	*P* value
*N*	28793	5804	4162	
Tumor size	36.566 ± 15.200	81.036 ± 8.339	129.538 ± 48.279	<0.001
Age	59.549 ± 12.389	61.090 ± 11.941	59.379 ± 11.794	<0.001
Age group				<0.001
<70 years	22567 (78.377%)	4375 (75.379%)	3351 (80.514%)	
≥70 years	6226 (21.623%)	1429 (24.621%)	811 (19.486%)	
Grade				<0.001
Grade I	3644 (12.656%)	296 (5.100%)	128 (3.075%)	
Grade II	16578 (57.576%)	2256 (38.870%)	1186 (28.496%)	
Grade III	7573 (26.302%)	2333 (40.196%)	1777 (42.696%)	
Grade IV	998 (3.466%)	919 (15.834%)	1071 (25.733%)	
N stage				<0.001
N0	28485 (98.930%)	5424 (93.453%)	3554 (85.392%)	
N1	308 (1.070%)	380 (6.547%)	608 (14.608%)	
M stage				<0.001
M0	28141 (97.736%)	4998 (86.113%)	3050 (73.282%)	
M1	652 (2.264%)	806 (13.887%)	1112 (26.718%)	
Bone metastasis				<0.001
No	28504 (98.996%)	5570 (95.968%)	3879 (93.200%)	
Yes	289 (1.004%)	234 (4.032%)	283 (6.800%)	
Brain metastasis				<0.001
No	28734 (99.795%)	5713 (98.432%)	4061 (97.573%)	
Yes	59 (0.205%)	91 (1.568%)	101 (2.427%)	
Liver metastasis				<0.001
No	28727 (99.771%)	5722 (98.587%)	3996 (96.012%)	
Yes	66 (0.229%)	82 (1.413%)	166 (3.988%)	
Lung metastasis				<0.001
No	28503 (98.993%)	5301 (91.334%)	3390 (81.451%)	
Yes	290 (1.007%)	503 (8.666%)	772 (18.549%)	
Insurance				<0.001
Uninsured	793 (2.754%)	197 (3.394%)	166 (3.988%)	
Insured	27636 (95.982%)	5547 (95.572%)	3959 (95.123%)	
Unknown	364 (1.264%)	60 (1.034%)	37 (0.889%)	
Marital status				0.095
Unmarried	9743 (33.838%)	1943 (33.477%)	1424 (34.214%)	
Married	17489 (60.740%)	3586 (61.785%)	2541 (61.052%)	
Unknown	1561 (5.421%)	275 (4.738%)	197 (4.733%)	
Fuhrman grade				<0.001
I	3631 (12.611%)	291 (5.014%)	126 (3.027%)	
II	16554 (57.493%)	2255 (38.853%)	1174 (28.208%)	
III	7558 (26.249%)	2296 (39.559%)	1736 (41.711%)	
IV	1050 (3.647%)	962 (16.575%)	1126 (27.054%)	
Surgery				<0.001
No	420 (1.459%)	159 (2.739%)	165 (3.964%)	
Partial nephrectomy	14474 (50.269%)	425 (7.323%)	139 (3.340%)	
Radical nephrectomy	13899 (48.272%)	5220 (89.938%)	3858 (92.696%)	
Race NEW				<0.001
Black	277 (0.971%)	52 (0.901%)	50 (1.210%)	
Others	1641 (5.755%)	342 (5.928%)	245 (5.929%)	
Unknown	3510 (12.310%)	516 (8.944%)	429 (10.382%)	
White	23086 (80.964%)	4859 (84.226%)	3408 (82.478%)	
T stage NEW				<0.001
1	25420 (88.285%)	548 (9.442%)	0 (0.000%)	
2	0 (0.000%)	2715 (46.778%)	1570 (37.722%)	
3	3299 (11.458%)	2403 (41.402%)	2293 (55.094%)	
4	74 (0.257%)	138 (2.378%)	299 (7.184%)	

**Table 2:** Univariate and multivariate logistic regression analyses of the risk factors for brain metastasis from renal cell carcinoma.

Exposure	Univariate	Multivariate
Age		
<70 years	1	1
≥70 years	0.802 (0.582, 1.105), 0.17707	0.653 (0.460, 0.927), 0.01719
T stage NEW		
1	1	1
2	12.116 (8.009, 18.329), <0.00001	2.656 (1.463, 4.823), 0.00133
3	11.820 (8.076, 17.300), <0.00001	2.303 (1.367, 3.881), 0.00173
4	44.218 (26.600, 73.505), <0.00001	2.215 (1.119, 4.384), 0.02245
Tumor size		
<4 cm	1	1
≥4 cm and <7 cm	5.240 (2.780, 9.880), <0.00001	2.270 (1.154, 4.467), 0.01755
≥7 cm and <10 cm	21.827 (11.950, 39.868), <0.00001	2.360 (1.128, 4.938), 0.02269
≥10 cm	34.081 (18.720, 62.047), <0.00001	2.070 (0.981, 4.365), 0.05602
Grade		
Grade I	1	1
Grade II	1.342 (0.690, 2.612), 0.38629	1.350 (0.650, 2.803), 0.42131
Grade III	3.397 (1.770, 6.520), 0.00024	1.772 (0.852, 3.684), 0.12578
Grade IV	10.877 (5.622, 21.044), <0.00001	2.462 (1.148, 5.281), 0.02066
N stage		
N0	1	1
N1	7.827 (5.738, 10.677), <0.00001	0.612 (0.423, 0.886), 0.00930
Bone metastasis		
No	1	1
Yes	23.869 (18.107, 31.466), <0.00001	3.313 (2.397, 4.578), <0.00001
Liver metastasis		
No	1	1
Yes	13.248 (8.498, 20.654), <0.00001	0.843 (0.505, 1.407), 0.51281
Lung metastasis		
No	1	1
Yes	50.854 (38.975, 66.354), <0.00001	10.882 (7.745, 15.289), <0.00001
Marital status		
Unmarried	1	1
Married	1.002 (0.770, 1.303), 0.98929	1.030 (0.771, 1.377), 0.84112
Unknown	0.517 (0.239, 1.119), 0.09390	0.878 (0.394, 1.956), 0.74979
Race NEW		
Black	1	1
Others	0.609 (0.225, 1.651), 0.32989	0.507 (0.170, 1.516), 0.22427
Unknown	0.151 (0.050, 0.454), 0.00075	0.135 (0.040, 0.452), 0.00118
White	0.526 (0.216, 1.284), 0.15839	0.429 (0.160, 1.153), 0.09347
Surgery		
No	1	1
Partial	0.004 (0.002, 0.008), <0.00001	0.042 (0.018, 0.101), <0.00001
Radical	0.058 (0.044, 0.077), <0.00001	0.132 (0.091, 0.193), <0.00001

**Table 3:** Interaction test between age and tumor size on the risk of brain metastases from renal cell carcinoma.

Age (years)	Tumor size (cm)	Crude	Model I	Model II
<70	<7	Ref.	Ref.	Ref.
≥70	<7	1.593 (0.915, 2.775), 0.1000	1.369 (0.780, 2.402), 0.2740	1.459 (0.837, 2.545), 0.1828
<70	≥7 and <10	9.583 (6.540, 14.042), <0.0001	3.787 (2.499, 5.740), <0.0001	6.805 (4.574, 10.124), <0.001
≥70	≥7 and <10	6.221 (3.482, 11.114), <0.0001	2.569 (1.391, 4.742), 0.0026	4.363 (2.416, 7.879), <0.001
<70	≥10	14.990 (10.336, 21.740), <0.0001	3.072 (2.006, 4.705), <0.0001	8.555 (5.709, 12.821), <0.001
≥70	≥10	8.252 (4.320, 15.761), <0.0001	2.159 (1.081, 4.314), 0.0293	4.868 (2.503, 9.470), <0.001
*P* interaction		0.0231	0.1679	0.0480

Model I was adjusted for bone metastasis, liver metastasis, and lung metastasis. Model II was adjusted for grade, N stage, marital status, race NEW, insurance, and Fuhrman grade.

## Data Availability

All data generated or analyzed during the present study was obtained from SEER program of the National Cancer Institute.
